# Imported* Plasmodium vivax* Malaria in the Russian Federation from Western Sub-Saharan Africa

**DOI:** 10.1155/2019/4610498

**Published:** 2019-02-26

**Authors:** Alla Baranova, Vladimir Sergiev, Lola Morozova, Natalia Turbabina, Evgeny Morozov

**Affiliations:** ^1^Martsinovsky Institute of Medical Parasitology, Tropical and Vector-borne Diseases, Sechenov University, 20, M.Pirogovskaya str., Moscow 119435, Russia; ^2^Department of Tropical Medicine and Parasitic Diseases, Sechenov University, 20, M.Pirogovskaya str., Moscow 119435, Russia; ^3^Department of Tropical, Parasitic Diseases and Disinfectology, Russian Medical Academy of Continuous Professional Education, Moscow, Russia

## Abstract

**Background:**

Imported cases of* Plasmodium vivax *malaria from western Africa are reported annually in the Russian Federation. Infected native African people moving from western Africa for different purposes (students, businessmen, specialists, etc.) or Russian citizens (tourists, diplomats, businessmen, etc.) incubate the pathogen until reaching their Russian destination.

**Methods:**

All imported and other confirmed malaria cases and the associated* Plasmodium *species recorded over the past twenty years throughout the Russian Federation were inventoried. These data were included in the national register. The data of imported malaria cases were analysed according to the species of* Plasmodium*, case origin, dates of importation, and patient nationality.

**Results:**

A total of 267* P. vivax-*infected patients who contracted the disease in western Africa were diagnosed in the Russian Federation from 1984 to 2017. Among them, 3 cases had mixed infections (2 with* P. vivax* +* P. falciparum* and 1* P. vivax* +* P. ovale*)*. Conclusion.* Our data reveal an existing risk of contracting* P. vivax* infections in towns of West sub-Saharan Africa despite the absence of local* P. vivax* infection records.

## 1. Background

*Plasmodium vivax *occupies the largest malaria-endemic region of the world, followed by* P. ovale* [1].* P. vivax *is almost absent in western sub-Saharan Africa, the epicentre of global malaria endemicity, due to genetic resistance of the local human population, which is in contrast with East Africa where* P. vivax* malaria is distributed mainly in Ethiopia, but infrequent further south too.* P. ovale* is distributed throughout sub-Saharan Africa and South-East Asia, and in the western Pacific. It is responsible for 1-5% of all malaria cases in West Africa [2].

There are several known genetic resistance mechanisms against* P. vivax.* One of them is Duffy negativity, which is present in western sub-Saharan African populations. The Duffy antigen is located on the surface of red blood cells. The protein encoded by this gene is a glycosylated membrane protein. It is general receptor but has some specificity for chemokines. This genetic aberration is the only one among malaria genetic protection mechanisms without known negative affects to the health of human beings. Duffy negativity as a susceptibility factor had been speculated from epidemiological data before its function was discovered in the 1970s [3]. Duffy negativity in West Africa is attributed to erythrocytes only. The Duffy antigen is not abolished from the membranes of other cells.

Nearly 100% of West Africans and their New World descendants, including the majority of Afro-Americans, have Duffy negativity in the form of heterozygotes; and the only place in the world with homozygotes for Duffy negativity are present. This genetic aberration is extremely rare in other ethnic groups. Duffy negativity is so effective in the protection against* vivax *malaria that several investigators in the United States failed to intentionally infect volunteers with* P. vivax *[4]. A recent investigation proved the homozygotes for Duffy negativity experienced total protection from* P. vivax*, and heterozygous individuals experienced partial protection [5].

Therefore,* P. vivax* almost disappeared from West Africa, but complete elimination was not achieved [6]. Despite the common belief of total* P. vivax* absence in West Africa and the lack of local records of the pathogen in western sub-Saharan countries, sporadic cases of* P. vivax* malaria imported from the region have been registered in countries where malaria has been eliminated [7]. Data on* P. vivax* malaria cases in West Africa are absent from WHO documents from the last 10 years [8, 9]. Sporadic cases of* P. vivax* malaria among Duffy negative subjects had been detected by PCR in West Cameroon recently [10]. Information regarding* P. vivax* malaria cases in Ethiopia, Eritrea, Somalia, Sudan, and Madagascar appeared in these documents, and some authors perceived the data as a recent development [5].

A confirmation of the presence of* P. vivax* in West Africa and imported cases of* P. vivax* malaria recorded from that region would be surprising. Unfortunately, the wheel is too often reinvented and history is continually rewritten when there is a clear set of events to look back on, to get to know better, and to understand [11]. The first systematic efforts to map the global endemicity of* P. vivax* were undertaken in 2012 only [12].

The aim of this paper is to call attention to an existing risk of contracting* P. vivax* infection, even during a short stay, in towns of West sub-Saharan Africa despite the absence of local records of* P. vivax* infection.

We present data regarding the importation of* P. vivax* malaria from West sub-Saharan Africa to the Russian Federation (1994-2017) and retrospective data (1984-1992) of the USSR.

## 2. Materials and Methods

The WHO Collaborating Centre for Research has been studying* vivax* malaria and operating at the Martsinkovsky Institute of Medical Parasitology & Tropical Medicine of the Ministry of Health Russian Federation in Moscow since 1982. In addition to scientific research, the centre conducts epidemiological monitoring and the evaluation of antimalarial measures [13].

Each diagnosed malaria case is required to be reported by any medical establishment in the Russian Federation to a local Rospotrebnadzor Department (State Sanitary and Epidemiological Organization) using the official form “Card of Epidemiological Investigation” that contains clinical, laboratory, entomological, and epidemiological information. In addition to reporting to local Rospotrebnadzor Department, thin and thick blood films together with a copy of the Card of Epidemiological Investigation for each identified malaria case are sent to the Reference Centre on Tropical Diseases at the Martsinkovsky Institute (Moscow) for microscopic confirmation by experts in malaria diagnoses. These data have been accumulated in the National Surveillance Register at the Martsinkovsky Institute since 1978. All imported cases are mapped to a country of origin.

The information derived from standard cards is entered into a database and analysed annually. The annual report presents data on the aggregate of cases from all parts of the Russian Federation. The reports are distributed to all 85 administrative territories of the country as analytical information.

The scope of malaria surveillance as a part of the general state surveillance system is as follows: malaria case reports are entered into a database; data is analysed and disseminated through an annual published analytical letter called “The Malaria Situation in the Russian Federation”; and planning, monitoring, and analysis of the efficacy of antimalarial measures are conducted. Malaria surveillance measures are carried out in all potential foci whenever imported malaria cases occur, as well as in new active foci when local transmission of malaria emerges. These measures include the following:

- detection of malaria patients
- epidemiological investigation of cases and foci
- entomological monitoring of breeding places and resting sites
- larviciding with ecologically safe methods (according to the transmission season)
- indoor residual spraying of pyrethroids (in cases of local transmission of malaria only)
- environmental measures, including hydroengineering and environmental control of breeding habitats
- medical personnel training
- health education of the foci population.

Data on registered cases of malaria in Russia are published in a table with all the reportable pathogens that are transmitted each year, produced by the CISID (WHO/EURO).

## 3. Results

The importation of* P. vivax* malaria to Russia from the countries of sub-Saharan Africa occurs almost annually by foreigners (students, businessmen) and Russian citizens (specialists on contracts, diplomats, sailors, and so on). According to the “National Register of Malaria in the Russian Federation” and the “Questionnaire of Epidemiological Survey of Malaria Foci,” 319 patients from sub-Saharan Africa with* P. vivax* malaria were identified from 1984 to 1992. Among them, 159 Russian citizens and foreigners were infected in 10 western African countries, with the majority infected in Nigeria (Table 1), and 160 patients were infected in eastern and Central Africa.

Apart from imported malaria cases in Russia, Dr. G. Suleymanov (Martsinkovsky Institute, former Senior Researcher) detected three Russian citizen patients with* P. vivax* malaria that had been working on contracts during his time in West Africa (1991-1992) [14]. Two of them had mixed* P. falciparum + P. vivax* infections (confirmed microscopically).

After the disintegration of the USSR, the number of travellers and persons working under contracts from the USSR/Russia decreased, and subsequently, the number of imported* P. vivax* malaria cases from West African countries to the Russian Federation decreased.

From 1994 to 2008,* 135* imported cases of* P. vivax* malaria from Africa were registered; among them, 104 were from West Africa [15], including 3 mixed infections: 2 -* P. falciparum + P. vivax *and 1 -* P. vivax + P. ovale* (rare combination of two types of tertian malaria) (Table 1). There were* 998* imported cases of* P. falciparum *malaria from Africa detected in the Russian Federation during these years, and among them,* 241* were from West Africa. One death attributable to malaria (*P. falciparum + P. ovale*) was reported in 2012 and occurred in a Russian resident visiting Sierra Leone.

All* P. vivax* malaria cases presented a fever, but none had severe and life-threatening conditions. All patients with* P. vivax* malaria received chloroquine for a 3-day period followed by a 14-day course of primaquine.

Only twenty subjects reported compliance with malaria chemoprophylaxis (mefloquine or atovaquone/proguanil). All of them had visited West Africa and did not complete the drug regimen. No history of individual chemoprophylaxis was available for the remaining cases.

**Figure 1:**
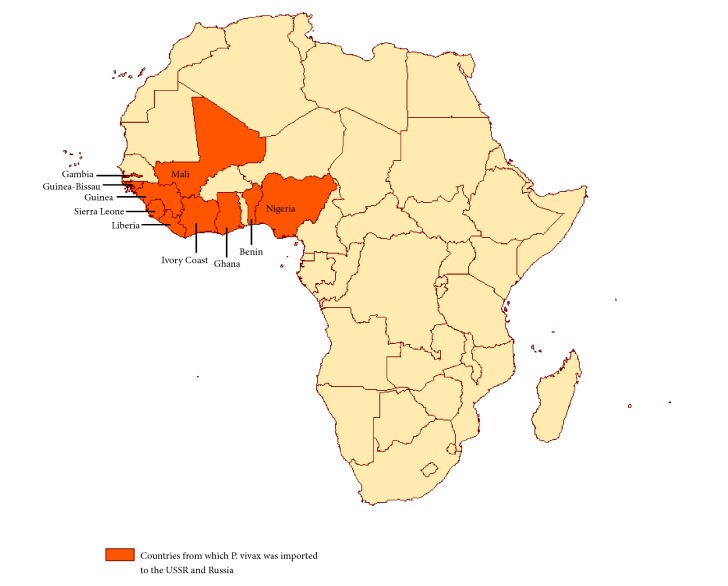
Countries of western Africa served as a source of imported* P. vivax* malaria to the USSR and Russia.

Imported* P. vivax* cases in 2009-2010 and 2013 originating in West African countries were not registered. One case from Guinea was recorded in 2011, one case from Cameroon was recorded in 2012, and two cases (Ivory Coast and Mali) were recorded in 2014. Imported* P. vivax* cases from West African countries were not registered in 2015-2017. Thus, the total number of imported malaria cases from western sub-Saharan African countries for 1984-2017 is 267. The West African countries that served as the source of imported malaria cases are presented in Figure 1.

## 4. Discussion

Imported cases of* P. vivax* to Russia from the countries of sub-Saharan Africa differed from those of the Newly Independent States (NIS) countries by clinical manifestation. Among the latter were a higher number of manifestations after long-term incubation of the pathogen (45% of imported cases from Tajikistan and 50% from Azerbaijan) [16].

Most imported cases of tertian malaria (*P. vivax*) by Russian citizens from sub-Saharan Africa presented the first clinical manifestation of the disease on the 12th-14th day after arrival in Russia. None of the imported* P. vivax* cases were followed by “introduced cases,” despite the susceptibility of endemic Russian mosquitoes to* P. vivax* from foreign countries [16].

The great majority of the cases (92.5%) were located in cities where the conditions for transmission were not favourable and medical care was easily accessible, i.e., early detection of patients was possible. In addition, the return of foreign students and Ph.D. students after their summer vacations occurs in October, when the transmission season of* P. vivax* by the mosquitoes* Anopheles maculipennis, An. messeae,* or* An. sacharovi* is over in the Russian territory.

The limitation of the study is travel histories of patients with* P. vivax* infection. Data of each case been collected for the last three years period, but we could not exclude that any individual presented his/her information incorrectly due to memory or other problems.

## 5. Conclusions

The results of the research analysis reveal an existing risk of contracting* P. vivax* infection in towns of West sub-Saharan Africa by local residents and foreigners despite the absence of local records of* P. vivax* infection. The appearance of imported cases of* P. vivax* malaria from that region of Africa should be considered in Russia and other countries.

This article can be used by epidemiologists to refine the global area of* P*.* vivax* distribution and by clinicians for the timely and correct diagnoses and treatment of malaria patients who arrive each year from African countries.

A current understanding of the spatial epidemiology and geographical distribution of* P. vivax* is far less developed than that for* P. falciparum*, representing a barrier to rational strategies for control and elimination of the disease. This communication may be considered important for studies on* P. vivax *distribution in sub-Saharan African countries.

## Figures and Tables

**Table 1:** Origin of imported *P. vivax* cases in the USSR and Russian Federation from 1984-2008.

Countries	Years								TOTAL
	USSR			Russian Federation					
	1984-1986	1987-1989	1990-1992	1994-1996	1997-1999	2000-2002	2003-2005	2006-2008	
Benin	7	14	5	0	0	0	0	0	*26*

Gambia	4	0	2	0	0	0	0	0	*6*

Ghana	3	10	5	0	3	3	6	2	*32*

Guinea	1	4	0	6	3	4	4	2	*24*

Guinea-Bissau	8	6	1	0	2	3	2	1	*23*

Ivory Coast	0	0	0	0	5	6	4	4	*19*

Liberia	0	0	0	0	0	0	1	2	*3*

Mali	4	12	8	0	8	6	2	0	*40*

Nigeria	21	23	11	2	2	3	2	2	*66*

Sierra Leone	1	3	6	3	3	2	4	2	*24*

*TOTAL*	*49*	*72*	*38*	*11*	*26*	*27*	*25*	*15*	*263*

## Data Availability

All documents and publications in Russian are available from the Archive and Library at the Martsinovsky Institute of the Sechenov University, Moscow, Russian Federation.
